# Amniotic membrane transplantation for neurotrophic corneal ulcers

**DOI:** 10.5935/0004-2749.2023-2022-0341

**Published:** 2024-02-23

**Authors:** Anna Carolina Badotti Linhares, Ana Caroline Martinelli, Mariela Regina Dalmarco Ghem, Paula Basso Dias, Daniel Wasilewski

**Affiliations:** 1 Departamento de Oftalmologia, Hospital de Clínicas da Universidade Federal do Paraná, Curitiba, PR, Brazil

**Keywords:** Amnion/transplantation, Corneal ulcer, Anterior eye segment, Keratitis

## Abstract

**Purpose:**

To evaluate the clinical results of cryopreserved amniotic membrane transplantation as a treatment option for refractory neurotrophic corneal ulcers.

**Methods:**

This prospective study included 11 eyes of 11 patients who underwent amniotic membrane transplantation for the treatment of refractory neurotrophic corneal ulcers at *Hospital de Clínicas da Universidade Federal do Paraná*, in the city of Curitiba, from May 2015 to July 2021. Patients underwent different surgical techniques in which the amniotic membrane was applied with the epithelium facing upward to promote corneal re-epithelialization.

**Results:**

The median age of the patients was 60 years (range, 34-82 years), and 64% were men. The predominant etiology of corneal ulcers was herpes zoster (45% of cases). Approximately one-third of the patients (27%) were chronically using hypotensive eye drops, and more than half (54%) had previously undergone penetrating corneal transplantation. At the time of amniotic membrane transplantation, 18% of the eyes had corneal melting, 9% had corneal perforation, and the others had corneal ulceration without other associated complications (73%). The time between clinical diagnosis and surgical treatment ranged from 9 days to 2 years. The corrected visual acuity was worse than 20/400 in 90% of the patients preoperatively, with improvement in 36% after 3 months of the procedure, worsening in 18% and remaining stable in 36%. Of the patients, 81% complained of preoperative pain, and 66% of them reported total symptom relief after the surgical procedure. In one month, 54.6% of the patients presented a closure of epithelial defect, and half of the total group evolved with corneal thinning. The failure rate was 45.5% of the cases.

**Conclusion:**

Cryopreserved amniotic membrane transplantation can be considered a good alternative for treating refractory neurotrophic corneal ulcers, as it resulted in significant improvement in pain (66%) and complete epithelial closure (60%) in many patients at 1 month postoperatively. Notably, the high failure rate highlights the need for further studies to identify patientand ulcer-related factors that may influence the outcomes of this procedure.

## INTRODUCTION

The long posterior ciliary nerves densely innervate the cornea and play a crucial role in maintaining epithelial integrity. Damage to this innervation compromises the protective reflex, reduces the number of stem cells and their metabolism, and disrupts, or decreases cellular mitoses^(1,2)^, thereby leading to a spectrum of vision--threatening corneal complications.

Neurotrophic ulcer is a degenerative disease of the corneal epithelium and stroma resulting from trigeminal innervation damage, which impairs corneal sensitivity, and triggers the aforementioned response. Herpetic keratitis, trauma, previous corneal surgery, diabetes mellitus, and neurosurgical procedures are common causes^(3,4)^.

Treating neurotrophic keratitis is challenging, as it aims to restore the tear film and improve corneal epithelial integrity while halting the progression and treating the lesion. Conventional therapy for this ulcer type includes using preservative-free artificial tears, eliminating toxic agents, tampon, or soft contact lens occlusion, tarsorrhaphy, conjunctival flap, and corneal transplantation^(5)^. Although these treatments can restore the corneal surface, they do not alter the pathological state, and many cases become refractory, demanding alternative therapies.

Amniotic membrane (AM) transplantation was first used in ophthalmic surgery in 1940, becoming popular only in 1990^(6)^. The AM emanates from the innermost part of the placenta and comprises a single epithelial layer, a thin basement membrane, and avascular stroma^(7)^. This structure has several properties, such as anti--adhesiveness, bacteriostaticity, injury protection, pain reduction, and epithelialization effect, besides having little antigenicity. AM transplantation is a promising treatment for corneal ulcers^(1)^.

AM transplantation stimulates epithelialization by acting as a basement membrane. Amniotic cells secrete multiple growth factors, such as keratinocyte, epidermal, and hepatocyte growth factors, which are involved in promoting corneal epithelium healing. The AM is also rich in neurotrophic factors, especially neuronal growth factor (NGF), which contributes to corneal nerve regeneration^(8)^. It signals mediators such as interleukins 1 and 2, antagonist receptors, pigment epithelium-derived factor, endostatin, and matrix metalloproteinase inhibitors. While the AM has an extracellular matrix rich in laminin, fibronectin, and collagen (I, II, and V) that serves as a substrate for limbal cell migration, the amnion can interfere with fibroblast maturity, thereby affecting inflammation, and angiogenesis^(3,9,10)^.

Inlay (graft), onlay (patch), and combined (sandwich) are key transplantation techniques. The first technique involves graft positioning in the injured area, with the epithelium facing up after defective tissue removal. The amnion acts as a substitute for injured tissue and is incorporated into the cornea. The number of layers used depends on the lesion depth. The second technique involves placing the epithelium over the periphery of the wound, facing downward, creating a mechanical barrier against environmental damage, symblepharon, and ankyloblepharon. The patch is later removed and not integrated into the cornea. The third technique synergizes the first two, where the graft provides structural integrity, and the patch protects the graft. The choice of technique varies depending on the ulcer depth, desired effect, and surgeon’s preference^(6)^.

This study evaluates the postoperative results of AM transplantation, such as changes in visual acuity (VA), symptom improvement, possible complications, and postsurgical refractoriness, to optimize the treatment of neurotrophic ulcers and provide further information about the use of amniotic membrane in their treatment.

## METHODS

The present study was approved by the Research Ethics Committee of *Hospital de Clínicas - Universidade Federal do Paraná* (HC -UFPR). All participants signed an informed consent form and had their data collected and stored under the ethical principles of privacy and confidentiality. This study was a prospective analysis of 11 patients who underwent AM transplantation for the treatment of neurotrophic ulcers at HC-UFPR between May 2015 and July 2021. All cases were characterized as Stage 3 Mackie’s classification, which refers to corneal ulcers with stromal involvement that may be complicated by stromal melting (two cases) or progression to corneal perforation (one case). Patients with insufficient preoperative data or who were lost to follow-up were excluded from the study.

VA was evaluated in all patients using Snellen’s original test, and lower VAs were converted to decimal and logMAR scales for statistical analyses, as follows: counting fingers, 1/100 (logMAR 2); hand motions, 1/200 (logMAR 2.3); light perception, 1/666 (logMAR 2.8); and amaurosis, 0 (logMAR 3).

The AM was obtained from patients who underwent elective and term cesarean delivery at the same hospital where the patients with ocular surface burn were referred to. Prior to AM collection, the parturient, and her companion provided their informed consent through a written consent form. The donors underwent laboratory analysis for HIV, hepatitis types B and C, and syphilis. These serologies were reconfirmed by analyzing umbilical cord blood after delivery. Positivity in any serology was an exclusion criterion for AM utilization.

Ophthalmologist residents prepared the placenta in a sterile operating room. The placenta was immersed in a diluted solution with gentamicin antibiotic and washed thoroughly with a 0.9% saline solution. The AM was then carefully separated from the corion by blunt dissection and flattened on sterile nitrocellulose filter paper, with the epithelium facing upward. Approximately 10 × 5 cm pieces were cut separately. The small AM samples were stored sterile at -80°C in a 1:1 ratio solution of glycerol and Dulbecco’s Modified Eagle Medium (Low glucose - 1,0 g/L) until used or discarded after six months.

Ophthalmologists performed all surgeries. Regarding the surgical technique, the AM graft was placed with the epithelium facing upward on the ocular surface and cornea. The stromal side was identified by noting stickiness. The AM was spread on the eye surface and cut to the appropriate size and shape, ensuring that the final piece size slightly exceeded the size of the defect to be covered. For the ulcer exceeding 5 mm, the membrane was placed over the hole cornea, after which it was sutured to the cornea periphery (1 mm from the limbus) using a single 10-0 Nylon continuous and linear suture all around the cornea, ensuring the needle reached 90% of the corneal depth in each bite and being careful not to perforate the cornea. This suture started and finished on the temporal side of the cornea, where the stitch was performed and buried through a circular movement of the hole suture. Following AM attachment to the cornea, another single running suture using 9-0 Vicryl was performed to attach the AM to the conjunctiva 3-4 mm away from the limbus. In smaller ulcers, the membrane was cut by ensuring that the final piece size slightly exceeded the size of the defect to be covered, and only the single 10-0 nylon continuous suture was placed to attach the AM. After surgery, a bandage contact lens (Acuvue bandage^®^) was put in place. When present, the Vicryl sutures were removed 2-3 weeks after surgery, and the nylon suture was removed around 2-4 months after surgery when complete membrane absorption had occurred.

Postoperatively, combined topical therapy with corticosteroids and antibiotics was instituted to reduce the inflammatory process and prevent secondary infection.

All patients included in this study had neurotrophic ulcers that were refractory to previously instituted clinical treatment. Patients with descemetocele or corneal perforation secondary to this etiology were also included in the study. In these patients, AM transplantation was performed using the same technique with the epithelium facing upwards in order to protect from outside infection, prevent more complications, and calm the eye until corneal transplantation. All participants continued in follow-up, with postoperative follow-up performed according to the needs of each case.

Statistical analysis was performed with Microsoft Excel 2000 and Graphpad Prism (Graphpad Prism for Windows 5.03). Spearman’s correlation was used for nonparametric data and the Mann-Whitney U test for nonparametric data with nominal components. Odds ratios were calculated for variables related to success along with their 95% confidence intervals. VA data were normalized to the logMAR scale, and a Wilcoxon signed-rank test was applied to analyze the relationship between the VAs before and after the intervention. A p-value <0.05 was considered statistically significant.

## RESULTS

Eleven eyes of eleven patients with a wide median age of 60 years (34-82 years) were included in this study (Table 1), and 7 (64%) patients were men. All patients had corneal ulcers despite previously instituted clinical treatments with artificial tears (11/11 [100%]), topical corticosteroids (10/11 [90%]), antivirals (5/11 [45%]), topical antibiotics (11/11 [100%]), and therapeutic contact lens (7/11 [63%]).

**Table 1:** Epidemiological characteristics, laterality, visual acuity preoperatively, and postoperatively

Case	Etiology	Presentation when indicated AM transplant	Age	Sex	Affected eye	Preoperative VA	Postoperative VA
1	Corneal toxicity from hypotensive eye drops	Neurotrophic ulcer with corneal perforation	60	Masculine	Right	Amaurosis	Amaurosis
2	HZV	Neurotrophic ulcer	82	Feminine	Right	20/160	20/400
3	HZV	Neurotrophic ulcer	75	Masculine	Right	Hands motion	20/200
4	HZV	Neurotrophic ulcer	47	Feminine	Right	Hands motion	Counting fingers
5	Facial palsy secundary to acustic neurinoma resection	Neurotrophic ulcer	36	Feminine	Right	Light perception	20/40
6	Bacterial keratitis	Neurotrophic ulcer	34	Masculine	Right	Hands motion	Hands motion
7	Acantamoeba keratitis	Neurotrophic ulcer with corneal melting	56	Masculine	Right	Hands motion	Light perception
8	HZV	Neurotrophic ulcer	82	Masculine	Right	Counting fingers	Counting fingers
9	Bacterial keratitis	Neurotrophic ulcer	73	Masculine	Right	Hands motion	20/400
10	HZV	Neurotrophic ulcer	69	Feminine	Right	Hands motion	Hands motion
11	Bacterial keratitis	Neurotrophic ulcer with corneal melting	53	Masculine	Right	Hands motion	Amaurosis

**Figure 1:**
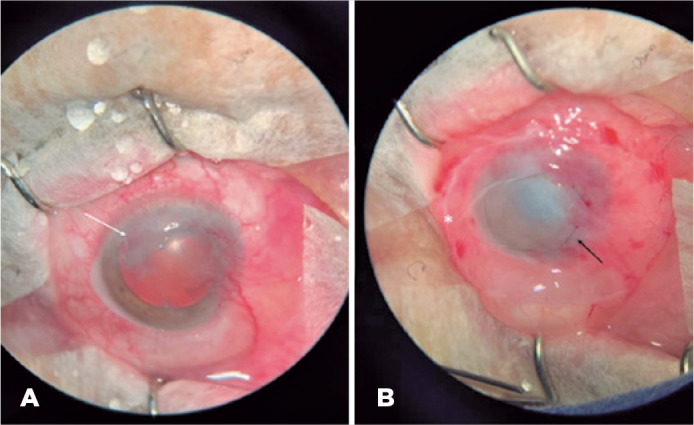
Preoperative and immediate postoperative AM transplantation in a neurotrophic ulcer secondary to ocular herpes zoster. A. Preoperative neurotrophic ulcer, indicated by the white arrow, secondary to ocular herpes zoster. B. Immediate postoperative AM transplantation; the asterisk shows the edges of the AM that can be seen in the image, and the black arrow indicates the continuous suture near the limbus.

The etiologies of neurotrophic ulcers found in this study were herpetic keratitis (5/11 [45%], Figure 1), bacterial infectious keratitis (3/11 [27%]), lagophthalmos secondary to facial paralysis after acoustic neuroma resection (1/11 [ 9%]), infectious amoeboid protozoan keratitis (1/11 [9%]), and corneal toxicity induced by hypotensive eye drops (1/11 [9%]). Of the 11 eyes, 3 (27%) were chronically using hypotensive eye drops for the treatment of glaucoma and 6 (54%) had already undergone penetrating corneal transplantation, with 1 of these eyes having both risk factors (9%). None of the patients had a previous diagnosis of diabetes.

**Figure 2:**
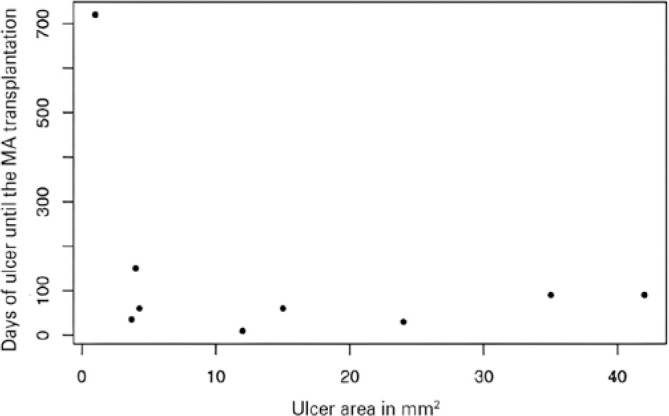
Time of evolution and ulcer size until AM transplantation.

Regarding the indications for AM transplantation, 2 (18%) patients had corneal melting, 1 (9%) had corneal perforation, and the others had corneal ulceration without other complications (73%). The mean area of the pre-transplant epithelial defect, stained with fluo-rescein, and visualized under cobalt light, was 14.7 square millimeters, ranging from small ulcers of 1 square millimeter to larger ulcers with an area corresponding to 42 square millimeters. The time between clinical treat-ment and surgical treatment ranged from 9 days to 2 years (Figure 2).

The prevalent laterality in our study was the right eye, which corresponded to 100% of the cases. Although some cases were observed in the left eyes, they were excluded due to incomplete clinical histories in medical records. Table 1 presents the preoperative VA of the patients, with the majority (10 [90%]) presenting VA worse than 20/400 on the Snellen table (among them, 7 = hand motions; 1 = light perception; 1 = counting fingers, and 1 = amaurosis) and one patient with an acuity of 20/160. VA 3 months after the procedure improved in 4 (36%) patients, worsened in 2 (18%), and remained stable in 4 (36%).

The main complaint of patients was pain, which was present in 9 (81%) patients preoperatively. Of these patients, 66% reported an improvement in total symptoms after the surgical procedure. One month after the operation, 54.4% of the patients had complete resolution of the epithelial defect.

**Figure 3:**
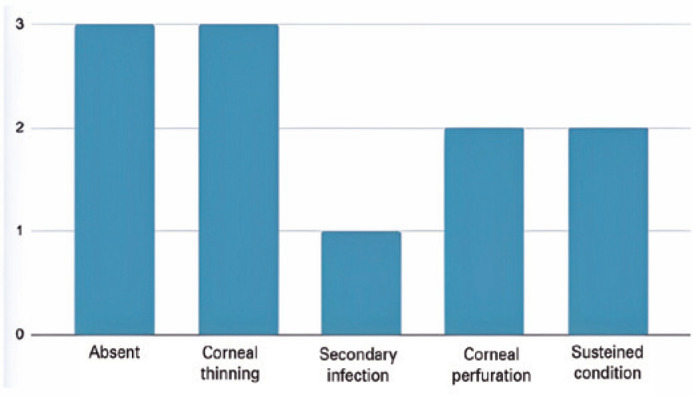
Postoperative complications.

Of the 6 (54.4%) eyes that had successful epithelial closure, half had no postoperative complications, while the other half evolved with corneal thinning. The failure rate, defined as the maintenance of the condition or worsening, occurred in 5 (45.6%) patients-one eye had an adjacent secondary infection and was submitted to conjunctival coverage, two progressed to corneal perforation in less than 1 month after surgery (one of them being submitted to a penetrating corneal transplant to preserve the eye that still had visual potential and the other eye to evisceration), and two eyes maintained the condition and were clinically treated 3 months after transplantation, when one had complete clinical improvement and the other underwent penetrating corneal transplantation associated with cataract surgery after persistent epithelial defect despite AM transplantation (Figure 3).

## DISCUSSION

AM transplantation is a surgical procedure for ocular surface reconstruction suitable for treating refractory neurotrophic ulcers, as it is relatively easy to learn and perform and it is more financially viable than other treatment options. Multilayer transplantation has been proposed for deeper ulcers^(11-13)^. All the cases analyzed arrived at the service center with a very advanced condition after previous treatments with conventional therapies. The predominant etiology was herpes, already described in the literature as the main cause of neurotrophic keratitis^(14)^.

Studies that compared conventional therapies (contact lens bandaging and tarsorrhaphy) and AM transplantation showed similar results between such approaches^(11-13,15)^. The present study showed that despite the rapid epithelial healing (16 days) observed, more than half of the patients required adjunctive therapy with tarsorrhaphy and therapeutic contact lenses (TCL), indicating the insufficiency of a single-layer AM for trea-ting severe cases of neurotrophic ulcer^(16,17)^. However, this effect may have occurred because treatment was initiated in cases that were already refractory to the aforementioned therapies and in advanced stages.

Five patients used TCL in the immediate postoperative period, and only one of them remained with open epithelium after combined therapy. Farias et al. compared TCL and AM utilization for corneal thinning and found significantly improved VA in membrane-treated patients, probably due to decreased stromal opacity^(18)^. In our study, these techniques were not compared. Notably, the function of the lens is to provide support for a firm AM attachment to the entire cornea and to protect the tissue and improve patient comfort.

Tarsorrhaphy was also used as a complementary treat-ment for a patient in this study who presented with central dellen, a transient shallow depression in the cornea near the limbus caused by local corneal stromal dehydration resulting from lagophthalmos. Combining tarsorrhaphy with AM treatment resulted in epithelial closure in this patient, who achieved the best VA (from light perception to 20/40 on the Snellen chart). However, a larger number of similar cases would be required to perform a statistical analysis of this association.

Two patients had complications that required additional treatment: a new AM coverage was required in one case, and conjunctival coverage combined with AM was required for complete epithelial closure in the other.

Mohan et al. used AM to treat infectious ulcers, an approach that was effective in reducing pain in the immediate postoperative period (p<0.001), congestion (p=0.003), and the need for corneal transplantation. The main complication reported was graft loss, and in these cases, a new AM transplant was performed^(19)^. In the present study, similar results were obtained regarding pain improvement, but divergent in relation to VA. This disparity is justifiable because more than half of the patients in this study had previously undergone corneal transplantation before the AM and two needed it in the postoperative period-factors that may have directly influenced VA.

Crisóstomo et al. evaluated AM transplantation in pediatric patients, realizing the complete success of the method in patients without limbal dysfunction. Only one case of treatment failure was observed (16.7%). Improved aesthetic appearance was observed in all patients analyzed, suggesting that young patients exhibit better aesthetic and functional results (VA) than advanced age patients^(20,21)^. Consistently, the wide median age of patients in the present study was 60 years, and 3 patients who were less than 50 years old evolved with epithelial closure and none of them showed worsening of vision.

The therapeutic efficacy of refractory neurotrophic ulcers with cryopreserved membrane transplantation is correlated with the regeneration of corneal innervation, resulting from the NGF, abundantly present in the amniotic tissue, evidenced by significantly increased density of the innervation of the cornea and its sensitivity^(22)^. However, despite their inflammation--suppressing effects, conventional topical anti-inflammatory therapies, such as cyclosporine, steroids, and nonsteroidal anti-inflammatory drugs, can increase the impairment of corneal innervation, thereby delaying its healing^(15)^. Because AM transplantation could help in nerve regeneration, its earlier application may yield even more favorable results, especially in environments in which access to more modern and efficient therapies are unavailable^(22)^.

AM can be considered a good alternative for the treat-ment of refractory neurotrophic ulcers, as evidenced by the improvement in pain observed in most eyes (66%) in this study as well as the complete epithelial closure in more than half of the patients (54.4%) in the first postoperative month. Studies with a greater number of patients and time of follow-up would be important for a better understanding of the associated factors. The potential benefits of using AM transplantation in milder cases could also be further investigated, especially considering the advantageous properties of AM compared to other therapeutic approaches, which may be less effective after the failure of initial treatments.
